# Mitochondria and pluripotency: from established models to emerging roles in adult stem cells

**DOI:** 10.3389/fbioe.2025.1654593

**Published:** 2025-09-03

**Authors:** Luminita Labusca, Camelia-Mihaela Zara-Danceanu

**Affiliations:** ^1^ Orthopedic and Trauma Clinic Emergency County Hospital Saint Spiridon, Iasi, Romania; ^2^ Magnetic Materials and Magnetic Nanosensors, National Institute of Research and Development for Technical Physics, Iasi, Romania

**Keywords:** adult pluripotent stem cells, embryonic stem cells, induced pluripotent stem cells, mitochondria, adult tissue stem cells

## Abstract

Pluripotency, once considered an exclusive attribute of early embryonic cells, is increasingly recognized in certain adult tissue-derived stem cell populations, challenging traditional developmental paradigms. Recent findings highlight mitochondria as key regulators of cellular identity, integrating metabolic status, redox signaling, and epigenetic cues to influence stemness and differentiation. This review synthesizes current knowledge on mitochondrial features (from morphology, dynamics, to bioenergetics and correlation to cellular epigenetic status) in pluripotent stem cells (ESCs and iPSCs) as well as in multipotent adult tissue stem cells (ASC) emphasizing transitions between glycolytic and oxidative metabolism during reprogramming and lineage specification. Particular attention is given to existing evidence on adult pluripotent-like stem cells, including VSELs, MAPCs, MUSE cells, MIAMI, and DFATs, which remain incompletely characterized but demonstrate promising regenerative capacities. While direct data on mitochondrial behavior in these cells are sparse, parallels with multipotent adult stem cells as well as with ESC and IPSCs suggest a model wherein stress-induced bioenergetic shifts, ROS signaling, and mitochondrial remodeling act as modulators of latent pluripotency. Understanding these mechanisms could offer insights on adult pluripotent stem cell role in orchestrating regeneration during major trauma or environmental stress as well as on their distinctive responsiveness compared to ASC. Such an approach could inform future strategies in regenerative medicine, offering novel insights into how adult cells might resume developmental plasticity through mitochondrial balance, intercellular transfer and networking.

## Introduction

Recent advances in biology have significantly reshaped our understanding of how cellular phenotypes are established, maintained, and transitioned. Contrary to earlier beliefs that cell identity is fixed, it is now evident that cells can alter their fate in response not only to developmental cues but also to metabolic fluctuations and environmental stressors encountered throughout post-developmental life (Sultan Sonia, 2017). Pluripotency exemplifies remarkable cellular plasticity, which is no longer presumed to be strictly associated with its developmental stages. Cellular pluripotency is defined by the unique capacity of a single cell type to differentiate into derivatives of all three embryonic germ layers (Yilmaz and Benvenisty, 2019).

Pluripotent stem cells (PSCs) can be derived from the embryo’s inner cell mass or reprogrammed *in vitro* and maintained in culture while preserving both their pluripotency and self-renewal capacity (Morgani et al., 2017). The establishment of embryonic stem cell (ESC) lines from mouse embryos (Evans and Kaufman, 1981) and the later development of induced pluripotent stem cells (iPSCs) represent landmark breakthroughs, recognized with Nobel Prizes in 2007 and 2012, respectively (Takahashi and Yamanaka, 2006; Thomson et al., 1998). The derivation of human equivalents of these cell types (Takahashi et al., 2007; Minger, 2013) marked a pivotal moment for both basic cell biology and the development of novel therapeutic strategies.

Engineered pluripotent cells have shown significant potential for cell-based therapies (Zahumenska et al., 2020), modeling human development and diseases, and creating *in vitro* personalized models for drug testing (Young et al., 2013). The existence of naturally occurring adult pluripotent stem cells has attracted considerable interest and controversy. While these cells are a compelling subject for basic, translational, and clinical research, their true pluripotency, reliable isolation, and therapeutic potential remain open questions (De Los Angeles et al., 2015). Understanding the mechanisms behind adult pluripotency and how cellular identity is maintained or changes after organismal maturation remains a debated issue in developmental biology, with significant implications for drug discovery and regenerative medicine.

To characterize *bona fide* pluripotent cells, four complementary perspectives have been proposed (Yilmaz and Benvenisty, 2019). The developmental perspective defines pluripotency based on cells arising naturally during embryogenesis, particularly within the inner cell mass of the pre-implantation blastocyst (naïve pluripotency) and the post-implantation epiblast (primed pluripotency) (Weinberger et al., 2016).

Cells are considered pluripotent if they exhibit both *in vitro* and *in vivo* differentiation potential into the three germ layers. The transcriptomic perspective focuses on gene expression and epigenetic profiles that typify the pluripotent state (Weinberger et al., 2016). A third, the reprogramming perspective, demonstrated that the introduction of a specific set of transcription factors (OCT4, SOX2, KLF4, and c-MYC) can revert differentiated somatic cells to a pluripotent state (Takahashi and Yamanaka, 2006; Takahashi et al., 2007). The fourth approach involves genome-wide functional screens to identify essential genes that are critical for maintaining pluripotency across species. These essential gene networks may serve as both diagnostic markers and experimental platforms to explore the regulatory machinery that sustains pluripotency (Yilmaz and Benvenisty, 2019).

In parallel with investigations into the genetic and epigenetic hallmarks of pluripotency, emerging evidence highlights the critical role of cellular energetics in supporting—and potentially inducing—the pluripotent state. The energy metabolism of eukaryotic cells is tightly linked to mitochondrial function. Mitochondrial morphology and dynamics, particularly during reprogramming, are now recognized as pivotal elements in determining cell fate. In PSCs, mitochondria are not merely powerhouses; they actively contribute to the maintenance of stemness, modulate differentiation pathways, and orchestrate metabolic rewiring. These features position mitochondrial dynamics as both a potential biomarker of pluripotency and a promising target for interventions aimed at reprogramming or regenerating tissues (Folmes et al., 2016).

This review begins by outlining the unique mitochondrial features of pluripotent cells, discussing specific characteristics of adult tissue-derived stem cells, and then delving into recent findings regarding energy metabolism in proposed adult pluripotent stem cells. We will further examine strategies aimed at modulating cell fate through metabolic intervention, highlighting the intersection between bioenergetics and cellular identity.

## The role of mitochondria in cell functioning-beyond the cellular powerhouse-

Mitochondria’s role extends beyond the classically described role of powerhouses of cells involved in generating energy from available nutrients. Aside from energy, mitochondria generate essential metabolic precursors required for the synthesis of lipids-fatty acids (FA), cholesterol, but also amino acids, glucose, heme, and nucleotides. Additionally, mitochondria produce metabolic by-products such as reactive oxygen species (ROS) and ammonia, which have both complex roles as secondary messengers and in the production of amino acids and nucleotides, respectively. Mitochondria employ specialized mechanisms to eliminate or repurpose other waste products (such as ethanol, glutamate, glutamine, and proline) (Spinelli and Haigis, 2018). Their role in orchestrating cellular adaptation to various external and internal stressors (nutrient scarcity, DNA damage, oxidative and endoplasmic reticulum (ER) stress) is closely related to cell fate from maintaining a functional cell *status quo* to contributing to cellular major shifts such as division, ageing and apoptosis, mitochondrial networks act as rapidly transitioning and dynamic modulators. In a systemic understanding, supra and intercellular mitochondrial networks could be involved in orchestrating local and/or systemic stress and adaptive responses at the cellular as well as at the organism and societal levels (Brand et al., 2013; Picard and Sandi, 2021).

Much of the understanding of the mitochondrial role in the pluripotent cellular state is derived from work dedicated to improving or/and streamlining nuclear reprogramming. It has become evident that the genetic and epigenetic alterations that allow an adult cell to revert to pluripotency are parallel by profound changes in mitochondrial number, morphology, and function to respond to sudden energy and metabolic increase. The transition of mitochondria during acquired pluripotency involves a complex interplay of metabolic, structural, and regulatory changes that are critical for obtaining and maintaining stem cell states as well as facilitating further differentiation processes. It is beyond the scope of this review to introduce these changes in detail; however, a brief overview will be presented for enhancing understanding.

## The metabolic shift: glycolysis replaces oxidative phosphorylation (OXPHOS)

IPSCs share metabolic similarities with ESCs and other rapidly proliferating cells, particularly in their reliance on glycolysis as the primary energy source, even under oxygen-rich conditions. This metabolic preference, known as the “Warburg effect,” supports rapid cell proliferation while limiting mitochondrial oxidative metabolism, thereby reducing oxidative stress (Prieto et al., 2020). Upon differentiation, mitochondrial maturation and structural remodeling drive a metabolic shift towards oxidative phosphorylation (OXPHOS).

Hypoxia-Inducible Factors (HIFs): HIF-1α stabilizes at low oxygen, further promoting glycolysis and suppressing mitochondrial respiration, thus maintaining pluripotency. Conversely, exposure to oxygen-rich environments degrades HIFs, reversing OXPHOS activation. IPSCs typically maintain their pluripotency and self-renewal abilities under hypoxic conditions (Hung et al., 2016), while high oxygen levels can trigger differentiation, reduce pluripotency, and even direct differentiation towards specific lineages (Hakim et al., 2014). Oxygen concentration and oxygen exposure have therefore been presented as master regulators of pluripotency, influencing fate, differentiation, as well as IPSC reprogramming efficiency (Nit et al., 2021).

Particularities in mitochondrial structure and dynamics in pluripotency and differentiation Pluripotent stem cells (PSCs) exhibit fragmented, perinuclear mitochondria with immature cristae, reflecting their limited contribution to cellular energy production. Upon differentiation, mitochondria undergo elongation, cristae maturation, and form interconnected networks, which enhance oxidative phosphorylation (OXPHOS) efficiency in parallel with lineage commitment.

Mitochondrial dynamics are governed by two opposing processes: fission—the division of mitochondria into smaller organelles, mediated mainly by dynamin-related protein 1 (DRP1) and its adaptors, which facilitates mitochondrial redistribution, quality control, and removal of damaged mitochondria. Fusion, -the merging of mitochondrial membranes to form elongated, interconnected networks-is driven by mitofusins (MFN1 and MFN2) on the outer membrane and optic atrophy protein 1 (OPA1) on the inner membrane (Zhong et al., 2019; Xu et al., 2013). These processes are tightly coordinated to maintain mitochondrial function, adapt to metabolic demands, and influence cell fate.

The balance between mitochondrial fission and fusion is critical for embryonic development, induced pluripotent stem cell (iPSC) reprogramming, and maintenance of the pluripotent phenotype. Excessive mitochondrial fusion increases cytosolic Ca^2+^ influx and activates calmodulin-dependent protein kinase II (CaMKII), leading to β-catenin degradation and impaired embryonic development, as demonstrated by tetraploid complementation assays (Zhong et al., 2019). In the early stages of reprogramming, activation of dynamin-related protein 1 (Drp1)—a key mediator of mitochondrial fission—facilitates efficient iPSC generation (Xu et al., 2013). Conversely, Drp1 inhibition disrupts cell cycle progression and induces G2/M phase arrest, impairing reprogramming efficiency (Prieto et al., 2016).

Mitofusin 2 (MFN2), a dynamin-like GTPase, and optic atrophy 1 (OPA1) regulate mitochondrial fusion and cristae structure. MFN2 downregulation or silencing has been reported to facilitate PSC differentiation into mesenchymal or neural lineages (Deng et al., 2022; Yi et al., 2020). OPA1, on the other hand, plays a role in maintaining the quiescence and activation potential of adult muscle stem cells (Baker et al., 2022). Taken together, pluripotency is characterized by a dominance of mitochondrial fission, whereas increased fusion, mitochondrial number, and intracellular networking typically parallel differentiation processes.

## Reactive oxygen species (ROS)

PSCs maintain low ROS levels due to their reliance on glycolytic metabolism. This low oxidative state is further supported by robust antioxidant defenses and efficient DNA repair mechanisms in both embryonic stem cells (ESCs) and iPSCs. Nonetheless, ROS generation is essential for somatic cell reprogramming, with both insufficient and excessive ROS impairing reprogramming efficiency (Zhou et al., 2016).

In human iPSCs, ROS levels fluctuate throughout the cell cycle, peaking during the S-to-G2/M transition. Exposure to antioxidants or reduced ROS levels in human ESCs and their differentiated fibroblast derivatives leads to decreased CYCLIN A expression, impaired S phase entry, accumulation of DNA damage, and activation of apoptotic pathways (Ivanova et al., 2021). Similarly, iron overload-induced ROS elevation significantly hampers proliferation in human ESCs and iPSCs via DNA damage, without affecting pluripotency (Ivanova et al., 2021).

## Mitochondria-mediated redox and epigenetic regulation in stem cell fate

Differentiation is often linked to elevated levels of reactive oxygen species (ROS) because of increased oxidative phosphorylation (OXPHOS) activity. ROS serve as signaling molecules that promote lineage specification (Han et al., 2020). Their role appears to be context-dependent, modulating differentiation outcomes; for example, ROS facilitates mesodermal differentiation of human embryonic stem cells (ESCs) via activation of p38 MAPK and AKT pathways (Ji et al., 2010), while elevated ROS levels impair endodermal differentiation of human induced pluripotent stem cells (iPSCs) by activating the tumor-associated transcription factor FOXC1. This impairment can be partially rescued by antioxidant treatment with N-acetylcysteine (NAC) (Oka et al., 2022).

Intrinsic variations in mitochondrial ROS (mitoROS) levels can influence nuclear redox balance and epigenetic regulation, such as histone H3 lysine 4 trimethylation (H3K4me3), thereby guiding cell fate during embryogenesis. Mouse ESCs with elevated mitoROS preferentially differentiate into mesoderm through primitive streak formation during gastrulation, whereas cells with low mitoROS favor neuroectodermal differentiation. This switch is regulated by the redox-sensitive transcription factor Nrf2 and is associated with the activation of the Wnt pathway. This switch is regulated by the redox-sensitive transcription factor Nrf2 and is linked to the activation of the Wnt pathway. Mitochondrial variability—both in number and in function-has been proposed to introduce extracellular “noise” that can override other sources of heterogeneity (e.g., asynchronous cell cycling), thereby influencing gene expression levels and potentially altering stem cell fate (Jhonston et al., 2025).

## Mitochondria in the epigenetic regulation of pluripotency and differentiation

Mitochondria are now recognized as key modulators of the epigenetic landscape, primarily through their roles in metabolite production, redox signaling, and organelle dynamics. These mitochondrial functions influence histone and DNA modifications, transcription factor activity, and chromatin remodeling.

Some mitochondrial metabolites act as essential cofactors or substrates for epigenetic enzymes. Notably, α-ketoglutarate (α-KG) supports the activity of DNA and histone demethylases, including ten-eleven translocation (TET) enzymes, DNA methyltransferases (DNMTs), and Jumonji-domain histone demethylases (JHDMs). Naïve mouse ESCs exhibit a high α-KG/succinate ratio, which maintains a demethylated chromatin state and supports pluripotency (Carey et al., 2015). Upon induction toward the trophoblast lineage, human ESCs undergo a metabolic shift that elevates α-KG levels, thereby promoting lineage conversion. This α-KG-driven metabolic reprogramming appears to function as a positive feedback loop by enhancing trophoblast specification and maturation (Van Nerum et al., 2025).

Sirtuins, a family of NAD^+^-dependent histone deacetylases, play a central role in linking cellular metabolism to epigenetic control of stemness and differentiation. Various isoforms with different subcellular localizations regulate the balance between maintaining pluripotency and committing to lineages.

SIRT1, a nuclear protein, modulates chromatin structure and transcription factor activity. It regulates the p53-mediated repression of the pluripotency gene NANOG. Conversely, SIRT1 downregulation activates developmental genes such as DLL4 (Delta-like ligand 4), TBX3 (T-box transcription factor 3), and PAX6 (paired box protein 6) (Calvanese et al., 2010). SIRT1 deficiency results in abnormal neural and glial differentiation in mESCs, underscoring its role in lineage specification (Mormone et al., 2023).

SIRT3 is located in the mitochondria and plays a role in mitochondrial stress responses and protecting cells from aging. It is essential for oocyte survival and quality, with SIRT3 deficiency leading to reduced blastocyst formation and increased oxidative stress. Enhancing SIRT3 expression may prevent age-related oocyte decline (Kordowitzki, 2024).

## Signalling pathways linking mitochondrial metabolism, maintenance, and pluripotency and differentiation

Multiple signaling pathways integrate mitochondrial function with pluripotency and stem cell differentiation, coordinating metabolic state, redox balance, and transcriptional networks.

AMP-activated protein kinase (AMPK), a sensor of cellular energy status activated under conditions of low ATP/high AMP, supports pluripotent stem cell (PSC) self-renewal by favoring glycolytic metabolism and suppressing OXPHOS. AMPK inhibits the mechanistic target of rapamycin (mTOR), which promotes anabolic processes and cell growth necessary for differentiation. Selective inhibition of mTORC1 has been demonstrated to enhance the self-renewal of hESCs and promote the development of the inner cell mass (Kim et al., 2024)

A functional axis linking SIRT1 and peroxisome proliferator-activated receptor alpha (PPAR-α/NR1C1), via PGC-1α, regulates mitochondrial biogenesis and methionine metabolism. Methionine depletion alters stemness in both normal and cancer stem cells, suggesting that targeting methionine metabolism may offer therapeutic strategies for regenerative medicine and cancer relapse (Siblini et al., 2022).

The Wnt/β-Catenin signaling pathway is crucial for maintaining pluripotency in both murine (mESCs) and human embryonic stem cells (hESCs), as well as in induced pluripotent stem cells (iPSCs). This pathway significantly enhances reprogramming efficiency (Marson et al., 2008). Activation of Wnt/β-Catenin promotes the self-renewal of pluripotent stem cells (Lee et al., 2025; Maurice and Angers, 2025) enabling them to continue dividing while remaining in an undifferentiated state. Wnt/β-catenin signaling is essential not only for pluripotency but also for stimulating differentiation, depending on the context and timing of activation. Transient Wnt activation can induce mesodermal differentiation in ESCs (Kreuser et al., 2020) and increase mesodermal commitment and cartilage tissue yield in human foreskin fibroblast-derived iPSCs (Davidson et al., 2012). In the hESC pathway, activation mediates neural crest formation (Leung et al., 2016). Timing and activation modes are, however, lineage specific. Constitutional Wnt pathway activation was found to block the multilineage differentiation potential in hematopoietic stem cells (HSC) (Kirstetter et al., 2006). Species (human versus mice) heterogeneity regarding Wnt pathway sensibility among ESC populations, as well as timing and duration of exposure, are all intricate factors demonstrating Wnt/β-Catenin involvement in cell fate decisions is not linear (Lien and Fuchs, 2014). Added to the complexity, the Wnt role in mitochondrial biogenesis and function could act as another pluripotency tuning mechanism. Evidence supports that mitochondrial retrograde signaling influences the Wnt pathway with effects on cell fate conversion. Mitochondria can regulate Wnt activation and β-catenin nuclear translocation, and the cell cycle activation mechanism is widely studied and targeted for its tumorigenesis implications (Delgado-Deida et al., 2020) that could influence pluripotency in cell fate decisions.

Notch signaling also modulates reprogramming efficiency, maintenance of stemness, and lineage differentiation in iPSCs (Osanathon and Egusa, 2022). Proteomic analyses show that Notch activation affects mitochondrial proteins involved in OXPHOS, the Krebs cycle, and fatty acid metabolism (Basak et al., 2014), pointing to a possible mechanistic link between Notch signaling and mitochondrial function. Moreover, coordinated mitochondrial dynamics during PSC differentiation appear to be governed through interactions among Notch, Wnt, and downstream YAP/TAZ signaling pathways (Lisowski et al., 2018).

## Pluripotency, mitophagy, and biogenesis

Mitochondrial biogenesis and autophagy coordination are critical for PSC functions. Mitochondrial biogenesis ensures an adequate supply of functional mitochondria to meet energy demands during differentiation and lineage commitment. Autophagy eliminates defective mitochondria, preventing the accumulation of reactive oxygen species (ROS) and maintaining cellular health. Even though in glycolytic predominant PSC, this process can have a lower impact, PSCs selectively degrade mitochondria via pathways like Parkin/PINK1 to maintain a glycolytic state. Differentiation, in turn, reduces mitophagy, allowing mitochondrial accumulation. Defective mitophagy results in the accumulation of depolarized mitochondria and compromises ESC self-renewal in hESCs in a PARK1/optineurin (OPTN) mediated mechanism (Wang et al., 2021). Mitophagy appears as an essential method of mitochondrial “quality control” during quiescence and pluripotency maintenance (Cairns et al., 2020). Mitophagy can also leverage mitochondrial biogenesis and increase OXPHOS during IPSC differentiation into endothelial lineages via mitochondrial phosphatase PGAM5 cleavage and consecutive PGC-1α-mediated transcriptional activation (Krantz et al., 2021).

Pharmacological blocking of mitochondrial fission and mitophagy dramatically impacts mIPSC reprogramming efficiency, pointing towards an important role in conversion to pluripotency as well as in the maintenance of pluripotent status (Vazquez-Martin et al., 2012).

mtDNA copy numbers are notoriously low in PSCs, reflecting a predominant glycolytic metabolism, increasing with differentiation, supporting OXPHOS. Recent evidence shows that the mitochondrial genome plays a broader role extending beyond ATP production. Variation in mtDNA, including haplotypes and copy number, can impact *in vitro* fertilization outcomes, embryo development, and tumorigenesis, respectively. These mtDNA features may also reflect an evolutionary adaptation. Crosstalk between the mitochondrial and nuclear genomes, including mtDNA’s influence on nuclear DNA methylation and gene expression during development, is increasingly recognized (St, 2016). Their perturbations during somatic cell reprogramming can not only impede process efficiency but also impact the stability of the resulting lineages (Haridhasapavalan et al., 2020).

## Adult stem cells and mitochondria

Unlikely, the PSCs that occur are transient during developmental stages or artificially “arrested” (ESCs) or engineered (IPSCs). Adult tissue stem cells (ASCs) are self-renewing clonogenic, multipotent elements located within specific tissues and organs throughout the body, residing in specialized microenvironments known as “niches.” ASCs are maintained in a quiescent state until they are activated by physiological needs, such as tissue turnover or repair after trauma or disease. ASCs are typically multipotent, meaning they can differentiate into a limited range of cell types pertinent to their tissue of origin. For example, hematopoietic stem cells (HSCs) can give rise to various blood lineages, mesenchymal stem cells (MSCs), to connective tissues (bone, cartilage, and adipose tissue), but typically not to cells from unrelated tissues. ASCs’ differentiation potential is generally restricted to the cell lineages of their specific embryonic layer/tissue of origin, even though exceptions might exist (Gonzalez and Bernad, 2012).

ASC mitochondrial metabolic features, dynamics, and mitophagy processes maintain similar stemness characteristics to PSC, displaying several particularities generated by their distinct phenotype shifts from dormancy to activation. ASC senses environmental changes that require exiting from quiescence and engaging in proliferative and differentiation stages. ASC mitochondria are kept in a relatively immature and low-activity state during quiescence to minimize ROS production, thus maintaining long-term stemness. As a general trait, low mTORC1 signaling in ASC minimizes mitochondrial ROS production to preserve quiescence (Mohammad et al., 2019). In several ASC types, discrete ROS fluctuation marks the exit from quiescence, a double-edged sword that exposes them to irreversible damage produced by high ROS levels generated by ageing, inflammation, or environmental toxicity.

HSCs in their hypoxic niche maintain low energy levels, relying mostly on glycolytic metabolism. Mitochondrial ROS production served as a subtle sensor in determining HSC fate. Distinct ROS elevation induces HSC differentiation and lineage commitment, reflected in the activation of the mammalian target of rapamycin (mTOR)-signaling pathway and increased mitochondrial biogenesis (Filippi and Ghaffari, 2019). Systemic increases in ROS levels during ageing, inflammation-induced ROS, or irradiation compromise HSC self-renewal and lineage commitment (Aires et al., 2021; Zhang et al., 2007) but are partially reversible by antioxidant administration (Hu et al., 2014).

MSCs were initially characterized retrospectively, based on their appearance in long-term tissue cultures, which limited precise definition of their native phenotype and anatomical distribution. Subsequent marker-based analyses showed that perivascular cells from diverse fetal and adult tissues share phenotypic and functional characteristics with bone marrow–derived MSCs, including clonal expansion and trilineage differentiation into osteogenic, chondrogenic, and adipogenic lineages—meeting the defining criteria for MSCs (Battula et al., 2007; Crisan et al., 2008a; Corselli et al., 2012). Further studies in retinal and brain pericytes were able to detected mitochondrial fragmentation, depolarization, and reduced oxidative metabolism under hyperglycemic stress (Trudeau et al., 2011) as well as bidirectional mitochondrial transfer with endothelial and glial cells in the neurovascular niche (Velmurugan et al., 2024). In contrast, no *in vivo* data are available on mitochondrial morphology or function in adventitial MSC-like cells, with existing work focusing primarily on phenotypic identity and lineage potential.

Adult hippocampal neural stem cells (NSC) activation is also linked to ROS fluctuations. Increased levels determine the exit from quiescent status, cellular proliferation, and lineage specification (Adusumilli et al., 2021). Exposure to heavy environmental metals such as cadmium, a potential neurotoxin, acts in part by inducing mitochondrial ROS increase in neural stem cells, activating oxidative stress and mitochondrial proton leak, compromising ATP production as well as NSC proliferative and differentiation ability (Luo et al., 2021).

Mitochondrial ROS is one of the factors that not only induces but is able to streamline lineage specification in MSCs, fine-tuning chondrogenic, osteogenic, or adipogenic lineage commitment (Li et al., 2017). Bone marrow adipose tissue (BMAT) imbalance during ageing, obesity, or metabolic syndrome and the associated increase in ROS within the bone marrow niche could be one of the factors implicated in osteopenia and age-related bone loss by compromising osteoblastogenesis versus adipose lineage conversion in the bone marrow MSC niche (Hardouin et al., 2016; Labusca, 2022). BMAT contains adipocytes with brown-like characteristics, including mitochondria enriched in uncoupling protein-1 (UCP1). Such mitochondria are abundant, display dense cristae, and have high respiratory capacity, enabling the dissipation of the proton gradient to generate heat rather than ATP through non-shivering thermogenesis. Similar brown adipocyte progenitors have also been identified in human skeletal muscle (Crisan et al., 2008b) suggesting that thermogenically competent adipocytes can arise from multiple perivascular niches.

Intestinal stem cells (ISC), being one of the most rapidly proliferative adult stem cell niches, display a high sensitivity to both external and internal mitochondrial ROS. The mouse (mISC)s respond to moderate ROS levels by entering the cell cycle, proliferation, and differentiation by means of P38 activation. Increased ROS levels generated by impaired mitochondrial metabolism and electron transport chain (ETC.) dysfunction are cleared by mitophagy. Mitophagy activity is strongly dependent on intestinal microbiota and nutrients by means of nucleotide-binding oligomerization domain-containing protein 2 (NOD2)-dependent activation of the autophagy-related gene ATG16L1 and is inhibited by calorie restriction, activated mTORC (Morris and Jasper, 2021). ATG16L1 genetic variants and consecutive mitophagy impairment are correlated with both Chron disease and several types of colon neoplastic proliferation centered on ISC anomalous activity (Cadwell et al., 2008).

Epidermal stem cell (ESC) progeny differentiation does not require mitochondrial ETC. However, it still relies on mitochondrial dynamics and biogenesis to enter proliferative stages, particularly after skin injury (Baris et al., 2011). Hair follicle stem cells (HFSC) activation and differentiation involve mitochondrial shift to aerobic respiration as well as increased levels of antioxidant mechanisms (such as superoxide dismutase and SOD2 expression) to maintain ROS balance (Tang et al., 2016).

Mitochondrial dynamics is another mechanism for regulating quiescence versus proliferation-differentiation. Increased fission and perinuclear arrangement correlate with the maintenance of stemness in ASC, while fusion is often associated with entering the cell cycle and proliferation (Spurlock et al., 2020). Expression of Mfn1, Mfn2, and OPA1 is generally involved in mitochondrial fusion; however, different ASC types may have particular activation mechanisms. Drp1-dependent mitochondrial fission is required for ASC asymmetric division (Katajisto et al., 2015) while its downregulation promotes differentiation (Fu et al., 2019).

Mitochondrial fission plays a role in satellite cell activation after muscle injury; meanwhile, excessive Drp1 and fission from mitochondrial 1 (Fis1), resulting in excessive mitochondrial fission, is activated via two specific ubiquitin ligases: MURF1 and atrogin-1. This activates the mitophagy process, resulting in the elimination of excessively fragmented mitochondria. Drp1 loss in aging can be responsive to age-related muscle atrophy (Hong et al., 2022). Loss of profusion protein Opa1 in the *Drosophila* NSC induces loss of neural progeny, possibly mediated by the role in mitochondrial cristae architecture (Dubal et al., 2022), while mutant Drp1 and consequent mitochondrial fusion decrease NSC proliferation in the cerebellum and hippocampal zones (Wakabayashi et al., 2009; Steib et al., 2014). Conversely, mutations in Mfn1 and Mfn2 that induce abnormal mitochondrial fragmentation are associated with decreased NSC proliferation in the hippocampus (Khacho et al., 2016) suggesting that the impact of proteins involved in mitochondrial fusion and fission interplay could have different impacts dependent on stem cell environment (*in vitro* versus *in vivo*) (Petrid et al., 2022) or anatomic location.

Selective degradation of damaged mitochondria via mitophagy is essential for preserving adult stem cell function, particularly given the fact that ROS-induced defective organelle targeting and clearance might be less effective. Increased mitophagy is largely associated with preserving stemness and dormancy, while mitochondrial biogenesis parallels proliferation and differentiation. Several mechanisms are involved in activating stem cell-specific mitophagy. Deletion of the autophagy gene Atg7 was found to increase mitochondria and ROS with increased proliferation as well as DNA damage in HSC, which, however, were proven dysfunctional and unable to restore hematopoiesis in irradiated mice (Mortensen et al., 2011).

HSCs were found to display enriched mitophagy-related genes, including Parkin, PTEN induced kinase (PINK1), optineurin (OPTN), outer mitochondrial membrane 7 (TOM7), microtubule-associated protein 1A/1B light chain 3a (AMPLC3a), and p62/sequestrome-1 (SQSTM1) (Ito et al., 2016). Excessive mitophagy and decreased mitochondrial biogenesis, however, prevent entering the differentiation stage in bone marrow MSCs from patients with the progressive nuclear palsy form of Parkinson’s disease, pointing towards an association of the disease with impaired balance of mitophagy/biogenesis (Angelova et al., 2018). In equine adipose-derived mesenchymal stem cells (ASCs) during the occurrence of metabolic syndrome, a mitophagy switch results in decreased chondrogenic potential and maintenance of stemness can point towards an adaptive mechanism to counteract metabolic ROS increase and therefore maintain stemness (Marycz et al., 2016).

Abnormal mitophagy induced by exposure to advanced glycation end products (AGEs) could induce bone marrow ASC(BMSC) senescence (Guo et al., 2021). Overexpression of mitochondrial NAD-dependent deacetylase SIRT3 can reduce AGE influence, decrease BMSC mitophagy and senescence associated with osteoporosis (Hu and Wang, 2022).

Metabolic reprogramming of quiescent ASC to OXPHOS metabolism is a well-known mechanism governing activation. That relies on epigenetic mechanisms. In satellite muscle cells, activation after intense activity or post-trauma relies on decreased intracellular NAD + levels and the histone deacetylase SIRT1, triggering H4K16 acetylation and activation of transcriptional activity (Walzik et al., 2023). In mice’ skeletal muscle satellite cells, genetic ablation of SIRT1 induced elevated H4K16 acetylation, resulting in deregulated myogenesis, reduced myofiber size, and impaired muscle regeneration (Ryall et al., 2015).

A rather low number, as well as mitochondrial connectivity and biogenesis, reliance on glycolysis are therefore features of pluripotent phenotypes, while multipotent cells residing in adult tissues appear to contain mitochondria with transitional features able to respond to environmental stimuli (Table 1).

**TABLE 1:** Mitochondrial features characteristic of pluripotent stem cells (embryonic stem cells–ESC, induced pluripotent stem cells–IPSc) and adult tissue stem cells (ASC).

Characteristics	Pluripotent stem cells (ESCs/iPSCs)	Multipotent stem cells (ASCs)	References
Mitochondrial morphology	Fragmented, perinuclear	Tubular, elongated	Folmes et al. (2016), Xu et al. (2013)
Cristae structure	Immature, poorly developed	Mature, dense	Brand et al. (2013), Picard and Sandi (2021), Prieto et al. (2020)
Metabolic profile	Glycolytic	Glycolytic/Oxidative	Hung et al. (2016), Hakim et al. (2014), Siblini et al. (2022)
Preferred energy pathway	Aerobic glycolysis (Warburg effect)	Aerobic glycolysis/OXPHOS	Hakim et al. (2014), Nit et al. (2021), Baker et al. (2022)
Mitochondrial dynamics	Dominant fission	Fission in quiescence, fusion upon activation of proliferation and differentiation status	Spinelli and Haigis (2018), Zhong et al. (2019), Xu et al. (2013)
ROS levels	Low	Moderate to high, dynamic	Zhou et al. (2016), Ivanova et al. (2021)
Mitochondrial biogenesis	Low	Induced upon activation	Ulfig and Jakob (2024), Carey et al. (2015)
Mitophagy activity	High (to maintain glycolytic state)	Moderate, supports quality control	Deng et al. (2022), Yi et al. (2020)
mtDNA copy number	Low	Higher than PSCs	Hung et al. (2016), Hakim et al. (2014), Zhong et al. (2019)
Sensitivity to oxidative stress	Low to moderate	High, especially with aging or stress	Xu et al. (2013), Deng et al. (2022)

## Mitochondrial transfer ASC rescuing and sensor mechanism

**FIGURE 1:**
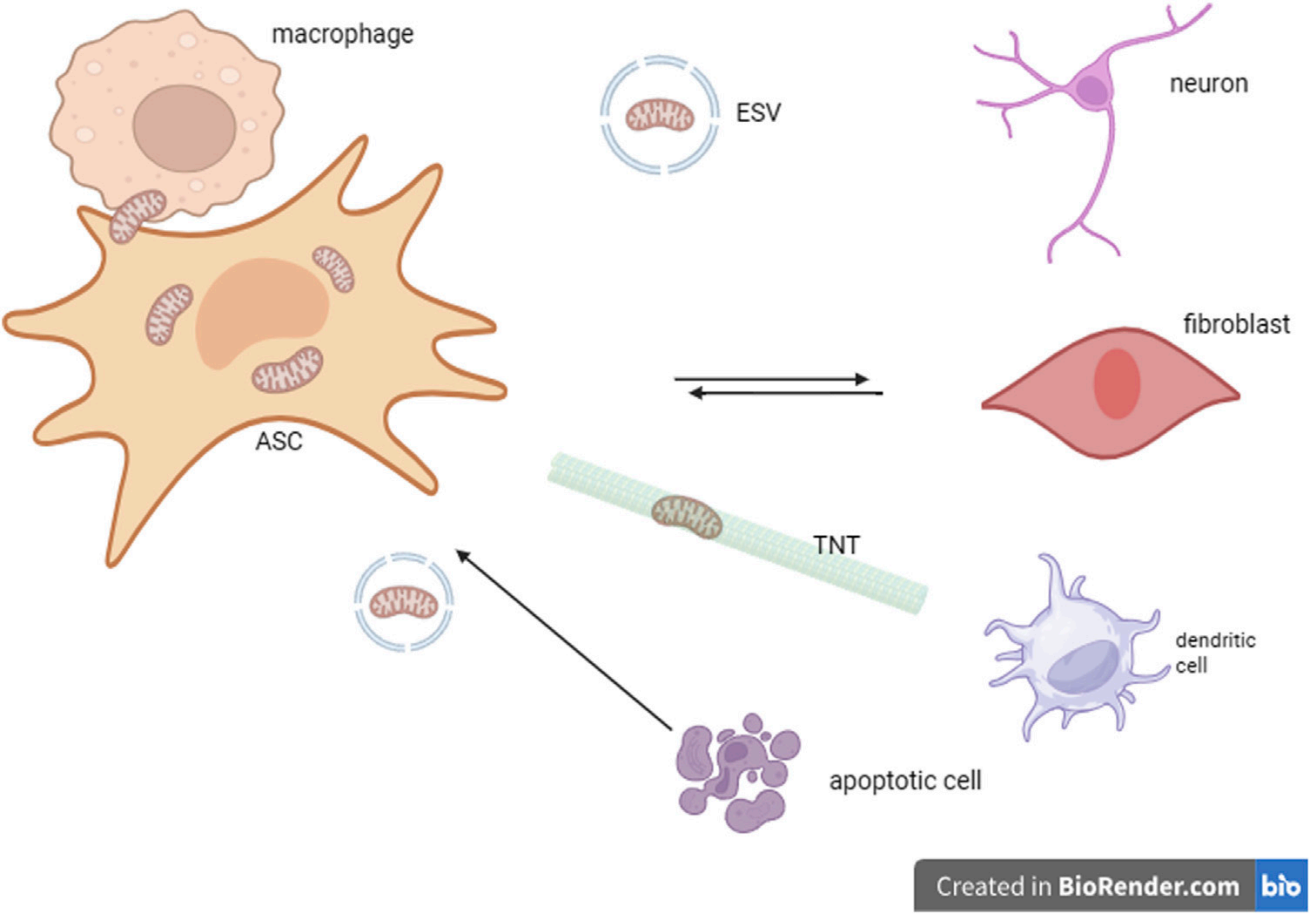
Bidirectional mitochondrial transfer between adult pluripotent stem cells (ASC), cells of the immune system (macrophages, dendritic cells), differentiated cells (represented by fibroblasts and neurons) and apoptotic cells " TNT= tunneling nanotubes: ESV- extracellular vesicles.

Intercellular mitochondrial transfer by means of tunnelling nanotubes (TNT) or extracellular vesicles has been recently described as a mode of intercellular communication that impacts tissue repair and regeneration, homeostasis, as well as tumor formation, metastasis, and ageing (Li et al., 2021). Mitochondrial transfer by ASC could be involved not only in cell bioenergetic rescue, mitochondrial quality control by means of mitophagy, but could also result from their potential role in sensing environmental stressors and in assisting tissue repair and regeneration (Rodriguez et al., 2018). In an *in vitro* study, human MSCs and skin fibroblasts have been shown to transfer mitochondria to alveolar epithelial cells with damaged mitochondria that cannot perform anaerobic respiration (Spees et al., 2006). In the following years, human bone marrow mesenchymal stem cells (BMMSCs) and human IPS-derived MSCs were shown to transfer *in vitro* mitochondria to cardiomyocytes, bronchial cells, corneas, as well as neurons damaged by diverse stressors such as ischemia and reperfusion (Han et al., 2016), oxygen deprivation (Li et al., 2014), or tobacco smoke exposure (Babenko et al., 2015). *In vitro* findings were confirmed *in vivo* in mice models of lung injury (Islam et al., 2012) or asthma (Yao et al., 2018) Fluorescence labeled human BMSCs (mito-DsRed) or GFP-labeled IPS derived MSCs respectively were tracked *in vivo* using live confocal microscopy and dual photon microscopy proving that mitochondrial transfer by TNT is involved in restoring OXPHOS in damaged tissues. MSC mitochondrial transfer to immune cells (such as macrophages, T-cells, and dendritic cells) could at least in part be involved in their immunomodulatory effect, demonstrated both *in vitro* (Jackson et al., 2016) and *in vivo* in rescuing bacterial-induced acute respiratory syndrome in mice (Koyanagi et al., 2005). We speculate that mitochondria transfer from immune or differentiated cells to MSCs could serve as a mechanism for influencing their fate. Bi-directional transfer from human MSC to renal tubular cells (Plotnikov et al., 2010) as well as TNT mediated transfer of healthy mitochondria to cells with blocked mitochondrial import function (Needs et al., 2024) demonstrate a mechanism of mitochondrial exchange that could be part of an adaptative mechanism. Even though such reports currently involve vitro experiments, future research could be performed to detect the eventual existence of an intercellular mitochondrial transfer as part of stress response. Mitochondria transfer from surrounding cells could possibly determine phenotype switching and induce cell cycle activation or differentiation possibly by favoring metabolic transition to OXPHOS, increasing the mitochondria’s number and ROS, or a combination of these factors. Mitochondrial trafficking from and to ASCs might be performed by alternative routes that have already been described (direct import, TNTs, extracellular vesicles) (Zhang et al., 2023). To facilitate both supportive and sensing functions, mitochondrial responses to stress may be triggered not only within individual cells but also communicated between tissues through the transfer of mitokines. This has important implications for maintaining homeostasis, responding to stress, and understanding processes related to aging and disease (Figure 1).

## Adult pluripotency -what is known and what is yet to be discovered-

Naturally occurring adult tissue–derived stem cells with reported pluripotent-like features—including very small embryonic-like stem cells (VSELs), multipotent adult progenitor cells (MAPCs), multilineage differentiating stress-enduring cells (MUSE), marrow-isolated adult multilineage inducible cells (MIAMI), and dedifferentiated fat cells (DFATs)—remain incompletely characterized, particularly with regard to their mitochondrial morphology, metabolic programming, and stress responsiveness. While these populations differ in origin, isolation methods, and stability of pluripotent traits, they share the potential for multilineage differentiation as well as the ability to activate under defined stress conditions. Certain of the so far isolated populations appear to persist *in vivo* in an ASC-like dormant state, whereas others may attain a pluripotent phenotype as an artifact of, or in response to, the isolation procedure itself. A summary of the so far described characteristics of adult pluripotent cells are listed in Table 2, To note, direct experimental data on mitochondrial morphology, bioenergetics, redox balance, and dynamics in adult pluripotent (-like) populations remain extremely limited. This scarcity probably reflects multiple factors: such as the rarity of these cells in native tissues, which constrains yield for functional assays; the heterogeneity introduced by non-standardized isolation methods. Finally, technical issues in applying high-resolution mitochondrial analyses—such as respirometry, single-cell imaging, or mitoROS quantification—to small, quiescent, and stress-sensitive populations may also be involved. As a result, much of our understanding is extrapolated from better-studied pluripotent and multipotent models, underscoring the need for systematic mitochondrial profiling in these rare adult-derived cells.

**TABLE 2:** Summary of the currently known characteristics of mitochondrial features in adult pluripotent stem cells.

Cell type	Mitochondrial morphology	Metabolic signature	Key stressors/Activators	Evidence for pluripotency
VSELs	Very few, spherical, perinuclear mitochondria; sparse ER; morphology consistent with glycolytic metabolism and deep quiescence	Predominantly glycolytic; low transcriptional activity	Hypoxia, ROS fluctuations; niche stress; possibly developmental origin from epiblast/PGCs	Expression of OCT4, NANOG, SSEA; differentiation into all three germ layers *in vitro*; controversy over isolation reproducibility
MAPCs	Not directly described; functional evidence of active mitochondria via mitochondrial transfer in rescue assays	Likely glycolytic (cultured in 5% O_2_); potential for rapid bioenergetic adaptation	Hypoxia during culture; growth factor exposure (PDGF, EGF)	Broad *in vitro* differentiation; cardiomyocyte rescue via mitochondrial transfer *in vivo*; non-immunogenic
MIAMI	Not described; long-term quiescence suggests low mitochondrial activity	Cultured in hypoxia; likely glycolytic	Hypoxia; culture conditions	Express OCT4, REX1; multipotent and some pluripotent markers; up to 50% population doubling without senescence
MUSE	Not described in detail; plausible stress-induced mitochondrial reprogramming	Quiescent under baseline; activated by stress; metabolism not directly studied	Severe stress (e.g., collagenase incubation), inflammation, ROS, hypoxia	Express NANOG, OCT3/4, TRA1-60; trilineage differentiation without teratoma formation; migratory and regenerative *in vivo*
DFATs	Not described; dedifferentiation may involve mitochondrial remodeling	Gene profile shift from lipid oxidation to glycolysis; upregulation of proliferation-related genes	Mechanical/culture stress (ceiling culture); dedifferentiation process	Transient expression of pluripotency factors (OCT4, SOX2, c-MYC, NANOG); trilineage differentiation *in vitro*; no teratoma formation

VSELs are a rare population isolated from adult bone marrow, peripheral blood, and other tissues. They express *bona fide* pluripotency markers (Oct4, Nanog, and SSEA), display primitive cell morphology, and demonstrate the ability to differentiate into derivatives of all three germ layers (Kucia et al., 2007). Even though a consistent number of laboratories have successfully isolated VSELs, others failed to do so, generating a still-existent controversy, much of which may be derived from applying non-homogeneous methods of isolation (Ratajczak et al., 2007) or from the scarcity of these cells within adult tissues, as well as from potentially overlapping populations differently nominated by groups using various methods of isolation (Suszynska et al., 2014). Such pluripotent elements could reside in adult tissues since embryo development, possibly derived from epiblast-derived stem cells (EPSC) and/or primordial germ cells (PG). The VSELs have around 2–4 μm diameter, a large nucleus with very few and spherical mitochondria and scattered ER, perhaps witnessing their glycolytic metabolism as well as low transcriptional activity as they reside in a highly quiescent state in bone marrow, gonads, and possibly other tissues (Ratajczak et al., 2019).

A population of multipotent adult bone marrow-derived progenitor cells (MAPCs), with important proliferation potential and extensive differentiation capabilities that are non-immunogenic, can be derived in large numbers with important regenerative medicine implications (Jacobs et al., 2013). Clinical grade MAPCs from bone marrow were proven to exert an immunomodulatory effect by inhibiting the cytotoxic effect of CD8-positive T cells (Plessers et al., 2016). Isolated mainly from bone marrow stroma, MAPCs differ from MSCs in terms of size and shape (trigonal and small cell body peak diameter 16 μm compared to more than 20 μm in MSCs) as well as characteristic culture conditions (that require hypoxia (5%O2)) alongside exposure to human-platelet-derived growth factor, PDGF, and human epidermal growth factor, EGF. (Khan and Newsome, 2019). Due to their hypoxic culture condition, they probably rely on glycolytic metabolism; however, evidence is scarce in this respect. Nevertheless, MAPCs were proven to exert therapeutic effects in rescuing cardiomyocytes *in vivo* in animal models of infarction employing mitochondrial transfer either by nanotubule or intercellular connexin-mediated transport. This evidence indirectly supports the existence of active and functional mitochondrial trafficking in MAPCs (Jameel et al., 2010).

Human MAPCs are non-immunogenic and exert potent immunomodulatory effects, influencing both innate and adaptive immune responses. These properties, together with their multilineage differentiation potential, have been reported in various preclinical models. While the original description of MAPCs by Verfaillie’s group (*Nature*, 2002) was retracted in 2024, independent studies have confirmed similar immunomodulatory phenotypes, supporting their relevance for regenerative and immunotherapeutic applications.

MIAMI (marrow isolated adult multilineage inducible cells) are derived from human bone marrow as cells with small body size, express multipotent as well as pluripotent surface cell markers (Oct-4 and Rex-1). MIAMI are cultivated in hypoxic conditions and can undergo up to 50% population doubling without signs of senescence and telomerase shortening (D'Ippolito et al., 2004). Given the remarkable similarity regarding cell morphology and surface markers as well as differentiation potential, it could be that they could represent a unique cell identity obtained by means of different isolation and/or cultivation methods. Remarkably, even if their long-term dormancy as well as “deep quiescence” has been recorded by several groups, little evidence exists on the mitochondrial metabolic and dynamic features that characterize this particular pluripotent phenotype.

Multilineage Differentiating Stress-Enduring (MUSE) cells have been isolated by several groups worldwide from the adult tissues of mammals and humans (adipose tissue, dermis, cord blood, bone marrow). MUSE cells can be isolated from cultured MSCs by stress exposure (such as incubation with collagenase solution) or through positive CD105/SSEA3 sorting (Heneidi et al., 2013). Currently, MUSE stem cells are considered to be naturally occurring pluripotent cells that reside in quiescence and are activated by severe stressing conditions *in vitro* or *in vivo* (Ossanna et al., 2023). Regardless of the tissue of origin, MUSE cells display markers of pluripotency (Nanog, OCT3/4, Tra1-60) as well as telomerase activity and asymmetric growth, being non-tumorigenic *in vitro* and not forming teratomas after being injected in mice (Kuroda et al., 2010). MUSE cells can undergo three embryonic layer differentiation (endoderm ectoderm and mesoderm) spontaneously or when exposed to specific conditions *in vitro* (Wakao et al., 2011). Muse cells are particular for being highly resistant to cellular stress (some methods of isolating them rely exactly on this ability), for having remarkable migratory and as well as tissue regenerative ability to integrate rapidly within highly damaged tissues (such as fulminant hepatitis, acute lung ischemia, diabetic skin ulcers, brain injury) (Iseki et al., 2017; Uchida et al., 2016; Kinoshita et al., 2015; Yabuki et al., 2018)

Current evidence supports that MUSE contribute to regeneration by paracrine signaling secretion of cytokines, growth factors, and extracellular vesicles—rather than trans differentiation, or engraftment contributing to modulation of inflammation, enhancement of endogenous progenitor activity, and improvement of the local microenvironment. Considering their high migratory and regenerative ability, such cells may belong to a highly conserved mechanism of cell survival, potentially compensating for lost organism regenerating ability in more evolutionary advanced animals, including mammals (Simerman et al., 2016).

To date, little has been reported on the mitochondrial features and metabolism of MUSE cells. Upon transplantation of human-derived MUSE cells in an immunodeficiency mouse model of focal brain injury, transplanted cells showed immunomarkers of the human Golgi apparatus and mitochondria (Uchida et al., 2017). Given MUSE cells’ quiescence, it is plausible that both inflammatory cytokines, ROS presence, and hypoxia could be sensed to induce mitochondrial metabolism reprogramming to initiate migration, proliferation, and further tissue-specific differentiation; however, this must be confirmed by further studies. (Trosko, 2018).

## Dedifferentiated fat cells (DFAT)

A surprising example of culture-induced transient pluripotency is represented by DFATs. By means of a ceiling culture, normal tissue-derived mature adipocytes undergo dedifferentiation and morphologic changes and transiently express pluripotency transcription factors (OCT4, SOX2, cMyc, NANOG), stage-specific embryonic antigens like SSEA3, as well as mesenchymal stem cell markers such as CD105. These cells can differentiate into several cell types from all three embryonic germ layers; however, these unlikely PSCs do not form teratoma after inoculation in immunodeficient mice (Jumabay and Boström, 2015). Profiling mature dedifferentiated adipocytes using microarray revealed that DFATS downregulates genes important for lipid metabolism and upregulates genes pertaining to cell proliferation, cell morphology, and differentiation (Ono et al., 2011). They have been tested for possible applications in several fields of regenerative medicine such as osteoporosis or replacing large bone defects in tissue engineering approaches (Shirakata et al., 2014; Kikuta et al., 2013), revascularization in ischemic tissues (Planat-Benard et al., 2004) and skin and soft-tissue regeneration (Asami et al., 2015), cartilage (Okita et al., 2015) or brain ischemic injuries (Kakudo et al., 2018). The main issues preventing broad adoption are mainly related to phenotype stability, culture standardization, and scaling up required for manufacturing (Liang et al., 2023). As in the other situation of adult-derived cells with pluripotent potential, no direct investigation or mention exists regarding mitochondrial metabolism and dynamics. The identified significant reductions in genes involved in lipid metabolism, including PDK4 (Pyruvate dehydrogenase kinase isozyme 4) – a key regulator of the pyruvate dehydrogenase complex, linking glycolysis to mitochondrial respiration. cLPL, FASN, LIPE, FABP4, and PPARG could suggest a metabolic shift away from fatty acid oxidation--or possibly toward glycolysis. Similarly, upregulation in genes involved in “Mitosis”, “M phase”, “cell cycle progression “functions is typically associated with a shift to glycolysis-dominant metabolism (“Warburg-like”) but this remains speculative unless further investigated. From another perspective, DFAT isolation and function could represent just another proof of cellular drastic phenotypic changes under stressful conditions that initiate profound adaptive responses of which mitochondrial metabolism and dynamics may be an important component.

Summarizing, there is currently little direct evidence for mitochondrial features in adult pluripotent stem cells (Table 2) Observations regarding their spatial morphology in VSELs as well as reduced number are indicative of a predominant fission-like dynamic while the extended periods of dormancy in VSELs, MUSE cells could indicate a predominant glycolytic and increased sensitivity to ROS as a signaling mechanism is a determinant of the exit out of dormancy as per parallelism with other adult stem cell populations. The case of DFATs invites us to explore the different situations of the potential shift from OXPHOS to transient glycolysis and the potential from fusion to fission, as well as their recovery after DFAT differentiation to various lineages. While understandable, the main focus has so far been on demonstrating adult pluripotent-like cells’ applicability to tissue regeneration, a deeper understanding of the mechanisms involved in the maintenance of pluripotency or acquisition of such traits will fuel further mechanistic understandings. Be they “awakened by stress” or “induced by (stressful) culture conditions”, adult pluripotent cells are very likely to rely on mitochondrial responses for their profound phenotypic alterations that require massive bioenergetic adaptations, ROS handling, and a rapid response to modified environments. Current understanding underlines the role of mitochondria in stress responses—from remodeling networks via fission/fusion, initiating quality control through mitophagy and UPR^mt, affecting energy output, and deciding cell fate (survival vs. apoptosis) (Xu et al., 2025).

Computational models of mitochondrial network response suggest that healthy mitochondria are poised at a critical point, balancing robustness with flexibility. Disrupting this balance through oxidative stress or excessive fusion decreases network complexity, impacting cellular functions like energy production, apoptosis regulation, or stress adaptation (Zamponi et al., 2018). Similar approaches could be undertaken to model and validate the role of mitochondrial metabolism, dynamics, and biogenesis in paralleling or maybe even orchestrating pluripotency in adult tissues.

Unlike ASC, adult pluripotent stem cells, mitochondria may be more capable of sensing environmental cues and switching from dormancy to rapid proliferative and differentiation phenotypes to orchestrate responses to major perturbations, rather than tissue turnover and minor stress. The role of intercellular and trans tissular mitochondrial networking in adaptive responses is yet unexplored.

## Concluding remarks

Mitochondria are increasingly perceived as dynamic regulators of cell identity, metabolic adaptation, and stress responsiveness. They are closely associated with but also extend their classical role as cell powerhouses. Their contribution to the acquisition, maintenance, and exit from pluripotent states—whether in embryonic, induced, or adult contexts—implies functions beyond ATP production. Across developmental and experimental settings, a consistent theme emerges that transitions in stemness states are paralleled by tightly regulated mitochondrial fission-fusion dynamics, mitophagy, and metabolic rewiring. Inviting hypotheses regarding intra-as well as extracellular networking capabilities, they are inviting further exploration. Such endeavors could possibly elucidate potential hierarchical systems coordination in both organogenesis, tissue maintenance, and repair as a result of external or internal perturbations.

Despite the compelling mechanistic insights gained from studies in ESCs and iPSCs, our understanding of mitochondrial roles in naturally occurring adult pluripotent-like cells remains fragmentary. VSELs, MAPCs, MIAMI, MUSE cells, and DFATs all challenge the traditional paradigm of lineage restriction in adult stem cells. However, direct evidence of their mitochondrial morphology, function, and metabolic programming is scarce. Whether these cells arise through various degrees of stress-induced reprogramming or represent a latent embryonic relic, their capacity for multilineage differentiation appears intimately tied to mitochondrial plasticity. Here again, possible mitochondrial sensing and networking functions could explain coordinated repair and regenerative mechanisms as well as their perturbations due to metabolic, inflammatory, senescent, or combinatorial origins.

A recurrent theme emerging from this review is the absence of direct mitochondrial analyses in naturally occurring adult pluripotent (-like) cell populations. While their regenerative capacity and stress responsiveness have been described, the lack of detailed bioenergetic, redox, and ultrastructural data leaves fundamental questions unanswered about how mitochondria contribute to the maintenance, activation, or loss of pluripotency in these rare cells. This lack of knowledge is partly methodologically generated by the extremely low yield of such cells, their heterogeneity across laboratories, and the technical challenges of adapting high-resolution mitochondrial assays to quiescent or stress-sensitive populations. However, in our understanding, it is also a conceptual issue, reflecting both the historical focus on proving pluripotency rather than probing its mechanistic underpinnings, and the partial abandonment of this field in favor of the engineered pluripotency and iPSC models. Addressing this gap will be essential to fully integrate adult pluripotent cells into the mitochondrial framework of stem cell biology. Future work should prioritize high-resolution bioenergetic profiling, single-cell mitochondrial imaging, mitoROS mapping, and integrated omics approaches coupled with lineage tracing to uncover how mitochondrial morphology, dynamics, and signaling intersect with the acquisition, maintenance, and functional deployment of pluripotency in adult tissues Addressing this gap will be essential to fully integrate adult pluripotent cells into the mitochondrial framework of stem cell biology, to deepen understanding of natural occurring regenerative processes and shifting cell states.

DFAT, VSEL, and other rare stem/progenitor populations have been detected *in vivo* in specific adult tissues, albeit at very low frequencies, and are generally expanded and characterized under *in vitro* conditions, where phenotypic drift may occur. Their contribution to endogenous repair processes remains uncertain, and their mitochondrial profile—a potential determinant of the stemness-to-differentiation transition—has not yet been elucidated. Defining this profile could provide important insights into their physiological relevance and inform strategies for their exogenous application in cell- or gene-based therapies.

We propose that future work should systematically characterize these cell populations using high-resolution bioenergetic profiling, mitochondrial imaging, redox state mapping, and lineage tracing. Combining single-cell omics and spatial biology with mitochondrial network topology and flux analysis can uncover regulatory layers that link cellular quiescence and activation, especially in the context of cell fate transitions.

From a regenerative medicine approach and a bold translational perspective, manipulating mitochondrial function offers a novel axis for enhancing adult pluripotent cell potency, stability, and therapeutic applicability. Moreover, mitochondria-derived biomarkers may eventually serve as indicators of latent regenerative potential in adult tissues or even predictors of tissue resilience to aging and disease.

As this field eventually advances, mitochondria may no longer be perceived as merely responders to cellular change, but concurrent orchestrators of stem cell identity, balancing adaptability with stability. The possible intercellular and trans-tissular mitochondrial networks that could orchestrate adaptive stress responses invite further conceptual and analytical investigation.

## References

[B1] Adusumilli V. S. Walker T. L. Overall R. W. Klatt G. M. Zeidan S. A. Zocher S. (2021). ROS dynamics delineate functional states of hippocampal neural stem cells and link to their activity-dependent exit from quiescence. Cell Stem Cell 28 (2), 300–314.e6. 10.1016/j.stem.2020.10.019 33275875 PMC7875116

[B2] Aires R. Porto M. L. de Assis L. M. Pereira P. A. N. Carvalho G. R. Côco L. Z. (2021). DNA damage and aging on hematopoietic stem cells: impact of oxidative stress in ApoE-/- mice. Exp. Gerontol. 156, 111607. 10.1016/j.exger.2021.111607 34715304

[B3] Angelova P. R. Barilani M. Lovejoy C. Dossena M. Viganò M. Seresini A. (2018). Mitochondrial dysfunction in Parkinsonian mesenchymal stem cells impairs differentiation. Redox Biol. 14, 474–484. 10.1016/j.redox.2017.10.016 29096320 PMC5680522

[B4] Asami T. Soejima K. Kashimura T. Kazama T. Matsumoto T. Morioka K. (2015). Effects of combination therapy using basic fibroblast growth factor and mature adipocyte-derived dedifferentiated fat (DFAT) cells on skin graft revascularisation. J. Plast. Surg. Hand Surg. 49 (4), 229–233. 10.3109/2000656x.2015.1020315 25744232

[B5] Babenko V. A. Silachev D. N. Zorova L. D. Pevzner I. B. Khutornenko A. A. Plotnikov E. Y. (2015). Improving the post-stroke therapeutic potency of mesenchymal multipotent stromal cells by cocultivation with cortical neurons: the role of crosstalk between cells. Stem Cells Transl. Med. 4, 1011–1020. 10.5966/sctm.2015-0010 26160961 PMC4542870

[B6] Baker N. Wade S. Triolo M. Girgis J. Chwastek D. Larrigan S. (2022). The mitochondrial protein OPA1 regulates the quiescent state of adult muscle stem cells. Cell Stem Cell 29 (9), 1315–1332.e9. 10.1016/j.stem.2022.07.010 35998642 PMC10249109

[B7] Baris O. R. Klose A. Kloepper J. E. Weiland D. Neuhaus J. F. Schauen M. (2011). The mitochondrial electron transport chain is dispensable for proliferation and differentiation of epidermal progenitor cells. Stem Cells 29 (9), 1459–1468. 10.1002/stem.695 21780252

[B8] Basak N. P. Roy A. Banerjee S. (2014). Alteration of mitochondrial proteome due to activation of Notch1 signaling pathway. J. Biol. Chem. 289 (11), 7320–7334. 10.1074/jbc.M113.519405 24474689 PMC3953249

[B9] Battula V. L. Bareiss P. M. Treml S. Conrad S. Albert I. Hojak S. (2007). Human placenta and bone marrow derived MSC cultured in serum-free, b-FGF-containing medium express cell surface frizzled-9 and SSEA-4 and give rise to multilineage differentiation. Differentiation 75 (4), 279–291. 10.1111/j.1432-0436.2006.00139.x 17288545

[B10] Brand M. D. Orr A. L. Perevoshchikova I. V. Quinlan C. L. (2013). The role of mitochondrial function and cellular bioenergetics in ageing and disease. Br. J. Dermatol 169 (Suppl. 2(0 2), 1–8. 10.1111/bjd.12208 23786614 PMC4321783

[B11] Cadwell K. Liu J. Brown S. Miyoshi H. Loh J. Lennerz J. K. (2008). A key role for autophagy and the autophagy gene Atg16l1 in mouse and human intestinal Paneth cells. Nature 456, 259–263. 10.1038/nature07416 18849966 PMC2695978

[B12] Cairns G. Thumiah-Mootoo M. Burelle Y. Khacho M. (2020). Mitophagy: a new player in stem cell biology. Biol. (Basel) 9 (12), 481. 10.3390/biology9120481 33352783 PMC7766552

[B13] Calvanese V. Lara E. Suárez-Alvarez B. Abu Dawud R. Vázquez-Chantada M. Martínez-Chantar M. L. (2010). Sirtuin 1 regulation of developmental genes during differentiation of stem cells. Proc. Natl. Acad. Sci. U. S. A. 107 (31), 13736–13741. 10.1073/pnas.1001399107 20631301 PMC2922228

[B14] Carey B. W. Finley L. W. Cross J. R. Allis C. D. Thompson C. B. (2015). Intracellular α-ketoglutarate maintains the pluripotency of embryonic stem cells. Nature 518 (7539), 413–416. 10.1038/nature13981 25487152 PMC4336218

[B15] Corselli M. Chen C. W. Sun B. Yap S. Rubin J. P. Péault B. (2012). The tunica adventitia of human arteries and veins as a source of mesenchymal stem cells. Stem Cells Dev. 21 (8), 1299–1308. 10.1089/scd.2011.0200 21861688 PMC3353742

[B16] Crisan M. Yap S. Casteilla L. Chen C. W. Corselli M. Park T. S. (2008a). A perivascular origin for mesenchymal stem cells in multiple human organs. Cell Stem Cell 3 (3), 301–313. 10.1016/j.stem.2008.07.003 18786417

[B17] Crisan M. Casteilla L. Lehr L. Carmona M. Paoloni-Giacobino A. Yap S. (2008b). A reservoir of brown adipocyte progenitors in human skeletal muscle. Stem Cells 26 (9), 2425–2433. 10.1634/stemcells.2008-0325 18617684

[B18] D'Ippolito G. Diabira S. Howard G. A. Menei P. Roos B. A. Schiller P. C. (2004). Marrow-isolated adult multilineage inducible (MIAMI) cells, a unique population of postnatal young and old human cells with extensive expansion and differentiation potential. J. Cell Sci. 117 (Pt 14), 2971–2981. 10.1242/jcs.01103 15173316

[B19] Davidson K. C. Adams A. M. Goodson J. M. McDonald C. E. Potter J. C. Berndt J. D. (2012). Wnt/β-catenin signaling promotes differentiation, not self-renewal, of human embryonic stem cells and is repressed by Oct4. Proc. Natl. Acad. Sci. U. S. A. 109 (12), 4485–4490. 10.1073/pnas.1118777109 22392999 PMC3311359

[B20] De Los Angeles A. Ferrari F. Xi R. Fujiwara Y. Benvenisty N. Deng H. (2015). Hallmarks of pluripotency. Nature 525 (7570), 469–478. 10.1038/nature15515 26399828

[B21] Delgado-Deida Y. Alula K. M. Theiss A. L. (2020). The influence of mitochondrial-directed regulation of Wnt signaling on tumorigenesis. Gastroenterol. Rep. (Oxf) 8 (3), 215–223. 10.1093/gastro/goaa025 32665853 PMC7333924

[B22] Deng L. Yi S. Yin X. Li Y. Luan Q. (2022). Downregulating MFN2 promotes the differentiation of induced pluripotent stem cells into mesenchymal stem cells through the PI3K/Akt/GSK-3β/Wnt signaling pathway. Stem Cells Dev. 31 (7-8), 181–194. 10.1089/scd.2021.0316 35088597

[B23] Dubal D. Moghe P. Verma R. K. Uttekar B. Rikhy R. (2022). Mitochondrial fusion regulates proliferation and differentiation in the type II neuroblast lineage in Drosophila. PLoS Genet. 18, e1010055. 10.1371/journal.pgen.1010055 35157701 PMC8880953

[B24] Evans M. J. Kaufman M. H. (1981). Establishment in culture of pluripotential cells from mouse embryos. Nature 292 (5819), 154–156. 10.1038/292154a0 7242681

[B25] Filippi M. D. Ghaffari S. (2019). Mitochondria in the maintenance of hematopoietic stem cells: new perspectives and opportunities. Blood 133 (18), 1943–1952. 10.1182/blood-2018-10-808873 30808633 PMC6497515

[B26] Folmes C. D. Ma H. Mitalipov S. Terzic A. (2016). Mitochondria in pluripotent stem cells: stemness regulators and disease targets. Curr. Opin. Genet. Dev. 38, 1–7. 10.1016/j.gde.2016.02.001 26953561 PMC5011451

[B27] Fu W. Liu Y. Yin H. (2019). Mitochondrial dynamics: biogenesis, fission, fusion, and mitophagy in the regulation of stem cell behaviors. Stem Cells Int. 2019, 9757201–9757215. 10.1155/2019/9757201 31089338 PMC6476046

[B28] Gonzalez M. A. Bernad A. (2012). Characteristics of adult stem cells. Adv. Exp. Med. Biol. 741, 103–120. 10.1007/978-1-4614-2098-9_8 22457106

[B29] Guo Y. Jia X. Cui Y. Song Y. Wang S. Geng Y. (2021). Sirt3-mediated mitophagy regulates AGEs-induced BMSCs senescence and senile osteoporosis. Redox Biol. 41, 101915. 10.1016/j.redox.2021.101915 33662874 PMC7930642

[B30] Hakim F. Kaitsuka T. Raeed J. M. Wei F. Y. Shiraki N. Akagi T. (2014). High oxygen condition facilitates the differentiation of mouse and human pluripotent stem cells into pancreatic progenitors and insulin-producing cells. J. Biol. Chem. 289 (14), 9623–9638. 10.1074/jbc.M113.524363 24554704 PMC3975012

[B31] Han H. Hu J. Yan Q. Zhu J. Zhu Z. Chen Y. (2016). Bone marrow-derived mesenchymal stem cells rescue injured H9c2 cells via transferring intact mitochondria through tunneling nanotubes in an *in vitro* simulated ischemia/reperfusion model. Mol. Med. Rep. 13 (2), 1517–1524. 10.3892/mmr.2015.4726 26718099 PMC4732861

[B32] Han Z. Xu Z. Chen L. Ye D. Yu Y. Zhang Y. (2020). Iron overload inhibits self-renewal of human pluripotent stem cells via DNA damage and generation of reactive oxygen species. FEBS Open Bio 10 (5), 726–733. 10.1002/2211-5463.12811 32053740 PMC7193162

[B33] Hardouin P. Rharass T. Lucas S. (2016). Bone marrow adipose tissue: to Be or not to Be a typical adipose tissue? Front. Endocrinol. (Lausanne) 7, 85. 10.3389/fendo.2016.00085 27445987 PMC4928601

[B34] Haridhasapavalan K. K. Raina K. Dey C. Adhikari P. Thummer R. P. (2020). An insight into reprogramming barriers to iPSC generation. Stem Cell Rev. Rep. 16 (1), 56–81. 10.1007/s12015-019-09931-1 31758374

[B35] Heneidi S. Simerman A. A. Keller E. Singh P. Li X. Dumesic D. A. (2013). Awakened by cellular stress: isolation and characterization of a novel population of pluripotent stem cells derived from human adipose tissue. PLoS One 8 (6), e64752. 10.1371/journal.pone.0064752 23755141 PMC3673968

[B36] Hong X. Isern J. Campanario S. Perdiguero E. Ramírez-Pardo I. Segalés J. (2022). Mitochondrial dynamics maintain muscle stem cell regenerative competence throughout adult life by regulating metabolism and mitophagy. Cell Stem Cell 29 (9), 1298–1314.e10. 10.1016/j.stem.2022.07.009 35998641

[B37] Hu S. Wang S. (2022). The role of SIRT3 in the osteoporosis. Front. Endocrinol. (Lausanne) 13, 893678. 10.3389/fendo.2022.893678 35692409 PMC9175005

[B38] Hu L. Cheng H. Gao Y. Shi M. Liu Y. Hu Z. (2014). Antioxidant N-acetyl-L-cysteine increases engraftment of human hematopoietic stem cells in immune-deficient mice. Blood 124 (20), e45–e48. 10.1182/blood-2014-03-559369 25287706 PMC4231425

[B39] Hung S. S. Van Bergen N. J. Jackson S. Liang H. Mackey D. A. Hernández D. (2016). Study of mitochondrial respiratory defects on reprogramming to human induced pluripotent stem cells. Aging (Albany NY) 8 (5), 945–957. 10.18632/aging.100950 27127184 PMC4931846

[B40] Iseki M. Kushida Y. Wakao S. Akimoto T. Mizuma M. Motoi F. (2017). Human muse cells, nontumorigenic phiripotent-like stem cells, have liver regeneration capacity through specific homing and cell replacement in a mouse model of liver fibrosis. Cell Transpl. 26 (5), 821–840. 10.3727/096368916X693662 27938474 PMC5657714

[B41] Islam M. N. Das S. R. Emin M. T. Wei M. Sun L. Westphalen K. (2012). Mitochondrial transfer from bone-marrow-derived stromal cells to pulmonary alveoli protects against acute lung injury. Nat. Med. 18 (5), 759–765. 10.1038/nm.2736 22504485 PMC3727429

[B42] Ito K. Turcotte R. Cui J. Zimmerman S. E. Pinho S. Mizoguchi T. (2016). Self-renewal of a purified Tie2+ hematopoietic stem cell population relies on mitochondrial clearance. Science 354 (6316), 1156–1160. 10.1126/science.aaf5530 27738012 PMC5164878

[B43] Ivanova J. S. Pugovkina N. A. Neganova I. E. Kozhukharova I. V. Nikolsky N. N. Lyublinskaya O. G. (2021). Cell cycle-coupled changes in the level of reactive oxygen species support the proliferation of human pluripotent stem cells. Stem Cells 39 (12), 1671–1687. 10.1002/stem.3450 34460135

[B44] Jackson M. V. Morrison T. J. Doherty D. F. McAuley D. F. Matthay M. A. Kissenpfennig A. (2016). Mitochondrial transfer via tunneling nanotubes is an important mechanism by which mesenchymal stem cells enhance macrophage phagocytosis in the *in vitro* and *in vivo* models of ARDS. Stem Cells 34 (8), 2210–2223. 10.1002/stem.2372 27059413 PMC4982045

[B45] Jacobs S. A. Pinxteren J. Roobrouck V. D. Luyckx A. van't Hof W. Deans R. (2013). Human multipotent adult progenitor cells are nonimmunogenic and exert potent immunomodulatory effects on alloreactive T-cell responses. Cell Transpl. 22 (10), 1915–1928. 10.3727/096368912X657369 23031260

[B46] Jameel M. N. Li Q. Mansoor A. Qiang X. Sarver A. Wang X. (2010). Long-term functional improvement and gene expression changes after bone marrow-derived multipotent progenitor cell transplantation in myocardial infarction. Am. J. Physiol. Heart Circ. Physiol. 298, H1348–H1356. 10.1152/ajpheart.01100.2009 20173039 PMC3774483

[B47] Jhonston I. G. Gaal B. Pires das Neves R. Enver T. Iborra F. Jones N. S. (2025). Cell Behavior (q-bio.CB); Biological Physics (physics.bio-ph) arXiv:1107.4499v2 [q-bio.CB]. 10.48550/arXiv.1107.4499

[B48] Ji A. R. Ku S. Y. Cho M. S. Kim Y. Y. Kim Y. J. Oh S. K. (2010). Reactive oxygen species enhance differentiation of human embryonic stem cells into mesendodermal lineage. Exp. Mol. Med. 42 (3), 175–186. 10.3858/emm.2010.42.3.018 20164681 PMC2845002

[B49] Jumabay M. Boström K. I. (2015). Dedifferentiated fat cells: a cell source for regenerative medicine. World J. Stem Cells 7 (10), 1202–1214. 10.4252/wjsc.v7.i10.1202 26640620 PMC4663373

[B50] Kakudo T. Kishimoto N. Matsuyama T. Momota Y. (2018). Functional recovery by application of human dedifferentiated fat cells on cerebral infarction mice model. Cytotechnology 70 (3), 949–959. 10.1007/s10616-018-0193-9 29352391 PMC6021288

[B51] Katajisto P. Dohla J. Chaffer C. L. Pentinmikko N. Marjanovic N. Iqbal S. (2015). Asymmetric apportioning of aged mitochondria between daughter cells is required for stemness. Science. 348 (6232), 340–343. 10.1126/science.1260384 25837514 PMC4405120

[B52] Khacho M. Clark A. Svoboda D. S. Azzi J. Maclaurin J. G. Meghaizel C. (2016). Mitochondrial dynamics impacts stem cell identity and fate decisions by regulating a nuclear transcriptional program. Cell Stem Cell 19, 232–247. 10.1016/j.stem.2016.04.015 27237737

[B53] Khan R. S. Newsome P. N. (2019). A comparison of phenotypic and functional properties of mesenchymal stromal cells and multipotent adult progenitor cells. Front. Immunol. 10, 1952. 10.3389/fimmu.2019.01952 31555259 PMC6724467

[B54] Kikuta S. Tanaka N. Kazama T. Kazama M. Kano K. Ryu J. (2013). Osteogenic effects of dedifferentiated fat cell transplantation in rabbit models of bone defect and ovariectomy-induced osteoporosis. Tissue Eng. Part A 19 (15–16), 1792–1802. 10.1089/ten.tea.2012.0380 23566022 PMC3700015

[B55] Kim J. K. Villa-Diaz L. G. Saunders T. L. Saul R. P. Timilsina S. Liu F. (2024). Selective inhibition of mTORC1 signaling supports the development and maintenance of pluripotency. Stem Cells 42 (1), 13–28. 10.1093/stmcls/sxad079 37931173 PMC10787279

[B56] Kinoshita K. Kuno S. Ishimine H. Aoi N. Mineda K. Kato H. (2015). Therapeutic potential of adipose-derived SSEA-3-positive Muse cells for treating diabetic skin ulcers. Stem Cells Transl. Med. 4 (2), 146–155. 10.5966/sctm.2014-0181 25561682 PMC4303359

[B57] Kirstetter P. Anderson K. Porse B. T. Jacobsen S. E. Nerlov C. (2006). Activation of the canonical Wnt pathway leads to loss of hematopoietic stem cell repopulation and multilineage differentiation block. Nat. Immunol. 7 (10), 1048–1056. 10.1038/ni1381 16951689

[B58] Kordowitzki P. (2024). Elucidating the role of sirtuin 3 in mammalian oocyte aging. Cells 13 (18), 1592. 10.3390/cells13181592 39329773 PMC11429517

[B59] Koyanagi M. Brandes R. P. Haendeler J. Zeiher A. M. Dimmeler S. (2005). Cell-to-cell connection of endothelial progenitor cells with cardiac myocytes by nanotubes: a novel mechanism for cell fate changes? Circ. Res. 96 (10), 1039–1041. 10.1161/01.RES.0000168650.23479.0c 15879310

[B60] Krantz S. Kim Y. M. Srivastava S. Leasure J. W. Toth P. T. Marsboom G. (2021). Mitophagy mediates metabolic reprogramming of induced pluripotent stem cells undergoing endothelial differentiation. J. Biol. Chem. 297 (6), 101410. 10.1016/j.jbc.2021.101410 34785214 PMC8661016

[B61] Kreuser U. Buchert J. Haase A. Richter W. Diederichs S. (2020). Initial WNT/β-Catenin activation enhanced mesoderm commitment, extracellular matrix expression, cell aggregation and cartilage tissue yield from induced pluripotent stem cells. Front. Cell Dev. Biol. 8, 581331. 10.3389/fcell.2020.581331 33195222 PMC7661475

[B62] Kucia M. Wu W. Ratajczak M. Z. (2007). Bone marrow-derived very small embryonic-like stem cells: their developmental origin and biological significance. Dev. Dyn. 236 (12), 3309–3320. 10.1002/dvdy.21180 17497671

[B63] Kuroda Y. Kitada M. Wakao S. Nishikawa K. Tanimura Y. Makinoshima H. (2010). Unique multipotent cells in adult human mesenchymal cell populations. Proc. Natl. Acad. Sci. U. S. A. 107 (19), 8639–8643. 10.1073/pnas.0911647107 20421459 PMC2889306

[B64] Labusca L. (2022). Adipose tissue in bone regeneration - stem cell source and beyond. World J. Stem Cells 14 (6), 372–392. 10.4252/wjsc.v14.i6.372 35949397 PMC9244952

[B65] Lee H. J. Lim S. H. Lee H. Han J. M. Min D. S. (2025). Phospholipase D6 activates Wnt/β-catenin signaling through mitochondrial metabolic reprogramming to promote tumorigenesis in colorectal cancer. Exp. Mol. Med. 57 (4), 910–924. 10.1038/s12276-025-01446-9 40259095 PMC12046002

[B66] Leung A. W. Murdoch B. Salem A. F. Prasad M. S. Gomez G. A. García-Castro M. I. (2016). WNT/β-catenin signaling mediates human neural crest induction via a pre-neural border intermediate. Development 143 (3), 398–410. 10.1242/dev.130849 26839343 PMC4760313

[B67] Li X. Zhang Y. Yeung S. C. Liang Y. Liang X. Ding Y. (2014). Mitochondrial transfer of induced pluripotent stem cell-derived mesenchymal stem cells to airway epithelial cells attenuates cigarette smoke-induced damage. Am. J. Respir. Cell Mol. Biol. 51 (3), 455–465. 10.1165/rcmb.2013-0529OC 24738760

[B68] Li Q. Gao Z. Chen Y. Guan M. X. (2017). The role of mitochondria in osteogenic, adipogenic and chondrogenic differentiation of mesenchymal stem cells. Protein Cell 8 (6), 439–445. 10.1007/s13238-017-0385-7 28271444 PMC5445026

[B69] Liu D. Gao Y. Liu J. Huang Y. Yin J. Feng Y. (2021). Intercellular mitochondrial transfer as a means of tissue revitalization. Signal Transduct. Target Ther. 6 (1), 65. 10.1038/s41392-020-00440-z 33589598 PMC7884415

[B70] Liang Z. He Y. Tang H. Li J. Cai J. Liao Y. (2023). Dedifferentiated fat cells: current applications and future directions in regenerative medicine. Stem Cell Res. Ther. 14 (1), 207. 10.1186/s13287-023-03399-0 37605289 PMC10441730

[B71] Lien W. H. Fuchs E. (2014). Wnt some lose some: transcriptional governance of stem cells by Wnt/β-catenin signaling. Genes Dev. 28 (14), 1517–1532. 10.1101/gad.244772.114 25030692 PMC4102759

[B72] Lisowski P. Kannan P. Mlody B. Prigione A. (2018). Mitochondria and the dynamic control of stem cell homeostasis. EMBO Rep. 19 (5), e45432. 10.15252/embr.201745432 29661859 PMC5934764

[B73] Luo H. Song B. Xiong G. Zhang B. Zuo Z. Zhou Z. (2021). Cadmium inhibits neural stem/progenitor cells proliferation via MitoROS-dependent AKT/GSK-3β/β-catenin signaling pathway. J. Appl. Toxicol. 41 (12), 1998–2010. 10.1002/jat.4179 33977565

[B74] Marson A. Foreman R. Chevalier B. Bilodeau S. Kahn M. Young R. A. (2008). Wnt signaling promotes reprogramming of somatic cells to pluripotency. Cell Stem Cell 3 (2), 132–135. 10.1016/j.stem.2008.06.019 18682236 PMC3235673

[B75] Marycz K. Kornicka K. Grzesiak J. Śmieszek A. Szłapka J. (2016). Macroautophagy and selective mitophagy ameliorate chondrogenic differentiation potential in adipose stem cells of equine metabolic syndrome: new findings in the field of progenitor cells differentiation. Oxid. Med. Cell Longev. 2016, 3718468. 10.1155/2016/3718468 28053691 PMC5178365

[B76] Maurice M. M. Angers S. (2025). Mechanistic insights into Wnt–β-catenin pathway activation and signal transduction. Nat. Rev. Mol. Cell Biol. 26, 371–388. 10.1038/s41580-024-00823-y 39856369

[B77] Minger S. L. (2013). Developing technologies to unlock the therapeutic and research potential of human stem cells. N. Biotechnol. 30 (4), 378–380. 10.1016/j.nbt.2012.11.006 23220475

[B78] Mohammad K. Dakik P. Medkour Y. Mitrofanova D. Titorenko V. I. (2019). Quiescence entry, maintenance, and exit in adult stem cells. Int. J. Mol. Sci. 20 (9), 2158. 10.3390/ijms20092158 31052375 PMC6539837

[B79] Morgani S. Nichols J. Hadjantonakis A. K. (2017). The many faces of Pluripotency: *in vitro* adaptations of a continuum of *in vivo* states. BMC Dev. Biol. 17, 7. 10.1186/s12861-017-0150-4 28610558 PMC5470286

[B80] Mormone E. Iorio E. L. Abate L. Rodolfo C. (2023). Sirtuins and redox signaling interplay in neurogenesis, neurodegenerative diseases, and neural cell reprogramming. Front. Neurosci. 17, 1073689. 10.3389/fnins.2023.1073689 36816109 PMC9929468

[B81] Morris O. Jasper H. (2021). Reactive Oxygen Species in intestinal stem cell metabolism, fate and function. Free Radic. Biol. Med. 166, 140–146. 10.1016/j.freeradbiomed.2021.02.015 33600942

[B82] Mortensen M. Soilleux E. J. Djordjevic G. Tripp R. Lutteropp M. Sadighi-Akha E. (2011). The autophagy protein Atg7 is essential for hematopoietic stem cell maintenance. J. Exp. Med. 208 (3), 455–467. 10.1084/jem.20101145 21339326 PMC3058574

[B83] Needs H. I. Glover E. Pereira G. C. Witt A. Hübner W. Dodding M. P. (2024). Rescue of mitochondrial import failure by intercellular organellar transfer. Nat. Commun. 15 (1), 988. 10.1038/s41467-024-45283-2 38307874 PMC10837123

[B84] Nit K. Tyszka-Czochara M. Bobis-Wozowicz S. (2021). Oxygen as a master regulator of human pluripotent stem cell function and metabolism. J. Pers. Med. 11 (9), 905. 10.3390/jpm11090905 34575682 PMC8466012

[B85] Oka S. Tsuzuki T. Hidaka M. Ohno M. Nakatsu Y. Sekiguchi M. (2022). Endogenous ROS production in early differentiation state suppresses endoderm differentiation via transient FOXC1 expression. Cell Death Discov. 8, 150. 10.1038/s41420-022-00961-2 35365611 PMC8976013

[B86] Okita N. Honda Y. Kishimoto N. Liao W. Azumi E. Hashimoto Y. (2015). Supplementation of strontium to a chondrogenic medium promotes chondrogenic differentiation of human dedifferentiated fat cells. Tissue Eng. Part A 21 (9–10), 1695–1704. 10.1089/ten.tea.2014.0282 25669848

[B87] Ono H. Oki Y. Bono H. Kano K. (2011). Gene expression profiling in multipotent DFAT cells derived from mature adipocytes. Biochem. Biophys. Res. Commun. 407 (3), 562–567. 10.1016/j.bbrc.2011.03.063 21419102

[B88] Osanathon T. Egusa H. (2022). Notch signaling in induced pluripotent stem cells Chapter 8 in in book: molecular Players in iPSC Technology. 10.1016/B978-0-323-90059-1.00003-8

[B89] Ossanna R. Veronese S. Quintero Sierra L. A. Conti A. Conti G. Sbarbati A. (2023). Multilineage-differentiating stress-enduring cells (muse cells): an Easily Accessible, pluripotent stem cell niche with unique and powerful properties for multiple regenerative medicine applications. Biomedicines 11 (6), 1587. 10.3390/biomedicines11061587 37371682 PMC10295618

[B90] Petridi S. Dubal D. Rikhy R. van den Ameele J. (2022). Mitochondrial respiration and dynamics of *in vivo* neural stem cells. Development 149 (23), dev200870. 10.1242/dev.200870 36445292 PMC10112913

[B91] Picard M. Sandi C. (2021). The social nature of mitochondria: implications for human health. Neurosci. Biobehav Rev. 120, 595–610. 10.1016/j.neubiorev.2020.04.017 32651001 PMC8058501

[B92] Planat-Benard V. Silvestre J. S. Cousin B. André M. Nibbelink M. Tamarat R. (2004). Plasticity of human adipose lineage cells toward endothelial cells: physiological and therapeutic perspectives. Circulation 109 (5), 656–663. 10.1161/01.cir.0000114522.38265.61 14734516

[B93] Plessers J. Dekimpe E. Van Woensel M. Roobrouck V. D. Bullens D. M. Pinxteren J. (2016). Clinical-grade human multipotent adult progenitor cells block CD8+ cytotoxic T Lymphocytes. Stem Cells Transl. Med. 5 (12), 1607–1619. 10.5966/sctm.2016-0030 27465071 PMC5189653

[B94] Plotnikov E. Y. Khryapenkova T. G. Galkina S. I. Sukhikh G. T. Zorov D. B. (2010). Cytoplasm and organelle transfer between mesenchymal multipotent stromal cells and renal tubular cells in co-culture. Exp. Cell Res. 316 (15), 2447–2455. 10.1016/j.yexcr.2010.06.009 20599955

[B95] Prieto J. León M. Ponsoda X. García-García F. Bort R. Serna E. (2016). Dysfunctional mitochondrial fission impairs cell reprogramming. Cell Cycle 15 (23), 3240–3250. 10.1080/15384101.2016.1241930 27753531 PMC5176137

[B96] Prieto J. Ponsoda X. Izpisua Belmonte J. C. Torres J. (2020). Mitochondrial dynamics and metabolism in induced pluripotency. Exp. Gerontol. 133, 110870. 10.1016/j.exger.2020.110870 32045634

[B97] Ratajczak M. Z. Machalinski B. Wojakowski W. Ratajczak J. Kucia M. (2007). A hypothesis for an embryonic origin of pluripotent Oct-4(+) stem cells in adult bone marrow and other tissues. Leukemia 21 (5), 860–867. 10.1038/sj.leu.2404630 17344915

[B98] Ratajczak M. Z. Ratajczak J. Kucia M. (2019). Very small embryonic-like stem cells (VSELs). Circ. Res. 124 (2), 208–210. 10.1161/CIRCRESAHA.118.314287 30653438 PMC6461217

[B99] Rodriguez A. M. Nakhle J. Griessinger E. Vignais M. L. (2018). Intercellular mitochondria trafficking highlighting the dual role of mesenchymal stem cells as both sensors and rescuers of tissue injury. Cell Cycle 17 (6), 712–721. 10.1080/15384101.2018.1445906 29582715 PMC5969546

[B100] Ryall J. G. Dell'Orso S. Derfoul A. Juan A. Zare H. Feng X. (2015). The NAD(+)-dependent SIRT1 deacetylase translates a metabolic switch into regulatory epigenetics in skeletal muscle stem cells. Cell Stem Cell 16 (2), 171–183. 10.1016/j.stem.2014.12.004 25600643 PMC4320668

[B101] Shirakata Y. Nakamura T. Shinohara Y. Taniyama K. Sakoda K. Yoshimoto T. (2014). An exploratory study on the efficacy of rat dedifferentiated fat cells (rDFATs) with a poly lactic-co-glycolic acid/hydroxylapatite (PLGA/HA) composite for bone formation in a rat calvarial defect model. J. Mater Sci. Mater Med. 25 (3), 899–908. 10.1007/s10856-013-5124-x 24363067

[B102] Siblini Y. Namour F. Oussalah A. Guéant J. L. Chéry C. (2022). Stemness of normal and cancer cells: the influence of methionine needs and SIRT1/PGC-1α/PPAR-α Players. Cells 11 (22), 3607. 10.3390/cells11223607 36429035 PMC9688847

[B103] Simerman A. A. Phan J. D. Dumesic D. A. Chazenbalk G. D. (2016). Muse cells: Nontumorigenic pluripotent stem cells present in adult tissues-A paradigm shift in tissue regeneration and evolution. Stem Cells Int. 2016, 1463258. 10.1155/2016/1463258 28070194 PMC5192335

[B104] Spees J. L. Olson S. D. Whitney M. J. Prockop D. J. (2006). Mitochondrial transfer between cells can rescue aerobic respiration. Proc. Natl. Acad. Sci. U. S. A. 103 (5), 1283–1288. 10.1073/pnas.0510511103 16432190 PMC1345715

[B105] Spinelli J. B. Haigis M. C. (2018). The multifaceted contributions of mitochondria to cellular metabolism. Nat. Cell Biol. 20 (7), 745–754. 10.1038/s41556-018-0124-1 29950572 PMC6541229

[B106] Spurlock B. Tullet J. Hartman J. L. Mitra K. (2020). Interplay of mitochondrial fission-fusion with cell cycle regulation: possible impacts on stem cell and organismal aging. Exp. Gerontol. 135, 110919. 10.1016/j.exger.2020.110919 32220593 PMC7808294

[B107] St J. J. C. (2016). Mitochondrial DNA copy number and replication in reprogramming and differentiation. Semin. Cell Dev. Biol. 52, 93–101. 10.1016/j.semcdb.2016.01.028 26827792

[B108] Steib K. Schäffner I. Jagasia R. Ebert B. Chichung Lie D. (2014). Mitochondria modify exercise-induced development of stem cell-derived neurons in the adult brain. J. Neurosci. 34, 6624–6633. 10.1523/jneurosci.4972-13.2014 24806687 PMC6608139

[B109] Sultan Sonia E. (2017). Developmental plasticity: re-conceiving the genotype Interface Focus.720170009. 10.1098/rsfs.2017.00009 PMC5566816 28839928

[B110] Suszynska M. Zuba-Surma E. K. Maj M. Mierzejewska K. Ratajczak J. Kucia M. (2014). The proper criteria for identification and sorting of very small embryonic-like stem cells, and some nomenclature issues. Stem Cells Dev. 23 (7), 702–713. 10.1089/scd.2013.0472 24299281 PMC3967357

[B111] Takahashi K. Yamanaka S. (2006). Induction of pluripotent stem cells from mouse embryonic and adult fibroblast cultures by defined factors. Cell 126 (4), 663–676. 10.1016/j.cell.2006.07.024 16904174

[B112] Takahashi K. Tanabe K. Ohnuki M. Narita M. Ichisaka T. Tomoda K. (2007). Induction of pluripotent stem cells from adult human fibroblasts by defined factors. Cell 131 (5), 861–872. 10.1016/j.cell.2007.11.019 18035408

[B113] Tang Y. Luo B. Deng Z. Wang B. Liu F. Li J. (2016). Mitochondrial aerobic respiration is activated during hair follicle stem cell differentiation, and its dysfunction retards hair regeneration. PeerJ 4, e1821. 10.7717/peerj.1821 27168957 PMC4860312

[B114] Thomson J. A. Itskovitz-Eldor J. Shapiro S. S. Waknitz M. A. Swiergiel J. J. Marshall V. S. (1998). Embryonic stem cell lines derived from human blastocysts. Science 282 (5391), 1145–1147. 10.1126/science.282.5391.1145 9804556

[B115] Trosko J. E. (2018). The role of the mitochondria in the evolution of stem cells, including MUSE stem cells and their biology. Adv. Exp. Med. Biol. 1103, 131–152. 10.1007/978-4-431-56847-6_7 30484227

[B116] Trudeau K. Molina A. J. Roy S. (2011). High glucose induces mitochondrial morphology and metabolic changes in retinal pericytes. Invest Ophthalmol. Vis. Sci. 52 (12), 8657–8664. 10.1167/iovs.11-7934 21979999 PMC3230288

[B117] Uchida H. Morita T. Niizuma K. Kushida Y. Kuroda Y. Wakao S. (2016). Transplantation of unique Subpopulation of fibroblasts, Muse cells, Ameliorates experimental stroke possibly via robust neuronal differentiation. Stem Cells 34 (1), 160–173. 10.1002/stem.2206 26388204

[B118] Uchida H. Niizuma K. Kushida Y. Wakao S. Tominaga T. Borlongan C. V. (2017). Human Muse cells Reconstruct neuronal circuitry in subacute lacunar stroke model. Stroke 48 (2), 428–435. 10.1161/STROKEAHA.116.014950 27999136 PMC5262965

[B119] Ulfig A. Jakob U. (2024). Redox heterogeneity in mouse embryonic stem cells individualizes cell fate decisions. Dev. Cell 59 (16), 2118–2133.e8. 10.1016/j.devcel.2024.07.008 39106861 PMC11338707

[B120] Van Nerum K. Wenzel A. Argemi-Muntadas L. Kafkia E. Drews A. Brun I. S. (2025). α-Ketoglutarate promotes trophectoderm induction and maturation from naive human embryonic stem cells. Nat. Cell Biol. 27, 749–761. 10.1038/s41556-025-01658-1 40269259 PMC12081308

[B121] Vazquez-Martin A. Cufi S. Corominas-Faja B. Oliveras-Ferraros C. Vellon L. Menendez J. A. (2012). Mitochondrial fusion by pharmacological manipulation impedes somatic cell reprogramming to pluripotency: new insight into the role of mitophagy in cell stemness. Aging (Albany NY) 4 (6), 393–401. 10.18632/aging.100465 22713507 PMC3409676

[B122] Velmurugan G. V. Vekaria H. J. Patel S. P. Sullivan P. G. Hubbard W. B. (2024). Astrocytic mitochondrial transfer to brain endothelial cells and pericytes *in vivo* increases with aging. J. Cereb. Blood Flow. Metab. 12, 0271678X241306054. 10.1177/0271678X241306054 39668588 PMC11638933

[B123] Wakabayashi J. Zhang Z. Wakabayashi N. Tamura Y. Fukaya M. Kensler T. W. (2009). The dynamin-related GTPase Drp1 is required for embryonic and brain development in mice. J. Cell Biol. 186, 805–816. 10.1083/jcb.200903065 19752021 PMC2753156

[B124] Wakao S. Kitada M. Kuroda Y. Shigemoto T. Matsuse D. Akashi H. (2011). Multilineage-differentiating stress-enduring (Muse) cells are a primary source of induced pluripotent stem cells in human fibroblasts. Proc. Natl. Acad. Sci. U. S. A. 108 (24), 9875–9880. 10.1073/pnas.1100816108 21628574 PMC3116385

[B125] Walzik D. Jonas W. Joisten N. Belen S. Wüst R. C. I. Guillemin G. (2023). Tissue-specific effects of exercise as NAD+ -boosting strategy: current knowledge and future perspectives. Acta Physiol. (Oxf) 237 (3), e13921. 10.1111/apha.13921 36599416

[B126] Wang C. Liu K. Cao J. Wang L. Zhao Q. Li Z. (2021). PINK1-mediated mitophagy maintains pluripotency through optineurin. Cell Prolif. 54 (5), e13034. 10.1111/cpr.13034 33931895 PMC8088463

[B127] Weinberger L. Ayyash M. Novershtern N. Hanna J. H. (2016). Dynamic stem cell states: naive to primed pluripotency in rodents and humans. Nat. Rev. Mol. Cell Biol. 17 (3), 155–169. 10.1038/nrm.2015.28 26860365

[B128] Xu X. Duan S. Yi F. Ocampo A. Liu G. H. Izpisua Belmonte J. C. (2013). Mitochondrial regulation in pluripotent stem cells. Cell Metab. 18 (3), 325–332. 10.1016/j.cmet.2013.06.005 23850316

[B129] Xu X. Pang Y. Fan X. (2025). Mitochondria in oxidative stress, inflammation and aging: from mechanisms to therapeutic advances. Signal Transduct. Target Ther. 10 (1), 190. 10.1038/s41392-025-02253-4 40500258 PMC12159213

[B130] Yabuki H. Wakao S. Kushida Y. Dezawa M. Okada Y. (2018). Human multilineage-differentiating stress-enduring cells exert pleiotropic effects to ameliorate acute lung ischemia-reperfusion injury in a rat model. Cell Transpl. 27 (6), 979–993. 10.1177/0963689718761657 29707971 PMC6050908

[B131] Yao Y. Fan X. L. Jiang D. Zhang Y. Li X. Xu Z. B. (2018). Connexin 43-mediated mitochondrial transfer of iPSC-MSCs alleviates asthma inflammation. Stem Cell Rep. 11 (5), 1120–1135. 10.1016/j.stemcr.2018.09.012 30344008 PMC6234920

[B132] Yi S. Cui C. Huang X. Yin X. Li Y. Wen J. (2020). MFN2 silencing promotes neural differentiation of embryonic stem cells via the Akt signaling pathway. J. Cell Physiol. 235 (2), 1051–1064. 10.1002/jcp.29020 31276200

[B133] Yilmaz A. Benvenisty N. (2019). Defining human pluripotency. Cell Stem Cell 25 (1), 9–22. 10.1016/j.stem.2019.06.010 31271751

[B134] Young H. E. Black A. C. Jr (2013). “Naturally occurring adult pluripotent stem cells,” in Reviews in cell biology and molecular medicine. Editor Meyers R. A. 10.1002/3527600906.mcb.201200017

[B135] Zahumenska R. Nosal V. Smolar M. Okajcekova T. Skovierova H. Strnadel J. (2020). Induced pluripotency: a powerful tool for *in vitro* modeling. Int. J. Mol. Sci. 21 (23), 8910. 10.3390/ijms21238910 33255453 PMC7727808

[B136] Zamponi N. Zamponi E. Cannas S. A. Billoni O. V. Helguera P. R. Chialvo D. R. (2018). Mitochondrial network complexity emerges from fission/fusion dynamics. Sci. Rep. 8 (1), 363. 10.1038/s41598-017-18351-5 29321534 PMC5762699

[B137] Zhang X. Sejas D. P. Qiu Y. Williams D. A. Pang Q. (2007). Inflammatory ROS promote and cooperate with the Fanconi anemia mutation for hematopoietic senescence. J. Cell Sci. 120 (Pt 9), 1572–1583. 10.1242/jcs.003152 17405815 PMC2857731

[B138] Zhang H. Li X. Fan W. Pandovski S. Tian Y. Dillin A. (2023). Inter-tissue communication of mitochondrial stress and metabolic health. Life Metab. 2 (1), load001. 10.1093/lifemeta/load001 37538245 PMC10399134

[B139] Zhong X. Cui P. Cai Y. Wang L. He X. Long P. (2019). Mitochondrial dynamics is critical for the full pluripotency and embryonic developmental potential of pluripotent stem cells. Cell Metab. 29 (4), 979–992.e4. 10.1016/j.cmet.2018.11.007 30527743

[B140] Zhou G. Meng S. Li Y. Ghebre Y. T. Cooke J. P. (2016). Optimal ROS signaling is critical for nuclear reprogramming. Cell Rep. 15 (5), 919–925. 10.1016/j.celrep.2016.03.084 27117405 PMC4856580

